# Mechanistically based blood proteomic markers in the TGF-β pathway stratify risk of hepatocellular cancer in patients with cirrhosis

**DOI:** 10.18632/genesandcancer.234

**Published:** 2024-02-01

**Authors:** Xiyan Xiang, Krishanu Bhowmick, Kirti Shetty, Kazufumi Ohshiro, Xiaochun Yang, Linda L. Wong, Herbert Yu, Patricia S. Latham, Sanjaya K. Satapathy, Christina Brennan, Richard J. Dima, Nyasha Chambwe, Gulru Sharifova, Fellanza Cacaj, Sahara John, James M. Crawford, Hai Huang, Srinivasan Dasarathy, Adrian R. Krainer, Aiwu R. He, Richard L. Amdur, Lopa Mishra

**Affiliations:** ^1^The Institute for Bioelectronic Medicine, The Feinstein Institutes for Medical Research and Cold Spring Harbor Laboratory, Division of Gastroenterology and Hepatology, Northwell Health, Manhasset, NY 11030, USA; ^2^Division of Gastroenterology and Hepatology, University of Maryland, Baltimore, MD 21201, USA; ^3^Department of Surgery, University of Hawaii, Honolulu, HI 96813, USA; ^4^Department of Epidemiology, University of Hawaii Cancer Center, Honolulu, HI 96813, USA; ^5^Department of Pathology, The George Washington University, Washington, DC 20037, USA; ^6^Department of Medicine, Sandra Atlas Bass Center for Liver Diseases and Transplantation, North Shore University Hospital/Northwell Health, Manhasset, NY 11030, USA; ^7^Office of Clinical Research, Northwell Health, Lake Success, NY 11042, USA; ^8^The Feinstein Institutes for Medical Research, Manhasset, NY 11030, USA; ^9^Institute of Molecular Medicine, The Feinstein Institutes for Medical Research, Manhasset, NY 11030, USA; ^10^Department of Medicine, Donald and Barbara Zucker School of Medicine at Hofstra/Northwell, Hempstead, NY 11549, USA; ^11^Hofstra Northwell School of Medicine, Hempstead, NY 11549, USA; ^12^Division of Gastroenterology and Hepatology, Cleveland Clinic, Cleveland, OH 44106, USA; ^13^Cold Spring Harbor Laboratory, Cold Spring Harbor, NY 11724, USA; ^14^Georgetown Lombardi Comprehensive Cancer Center, Washington, DC 20007, USA; ^15^Quantitative Intelligence, The Institutes for Health Systems Science, The Feinstein Institutes for Medical Research, Manhasset, NY 11030, USA; ^16^Department of Surgery, The George Washington University, Washington, DC 20037, USA; ^*^These authors contributed equally to this work

**Keywords:** TGF-β, hepatocellular carcinoma, myostatin, pyruvate kinase M2, biomarker

## Abstract

Hepatocellular carcinoma (HCC) is the third leading cause of death from cancer worldwide but is often diagnosed at an advanced incurable stage. Yet, despite the urgent need for blood-based biomarkers for early detection, few studies capture ongoing biology to identify risk-stratifying biomarkers. We address this gap using the TGF-β pathway because of its biological role in liver disease and cancer, established through rigorous animal models and human studies. Using machine learning methods with blood levels of 108 proteomic markers in the TGF-β family, we found a pattern that differentiates HCC from non-HCC in a cohort of 216 patients with cirrhosis, which we refer to as TGF-β based Protein Markers for Early Detection of HCC (TPEARLE) comprising 31 markers. Notably, 20 of the patients with cirrhosis alone presented an HCC-like pattern, suggesting that they may be a group with as yet undetected HCC or at high risk for developing HCC. In addition, we found two other biologically relevant markers, Myostatin and Pyruvate Kinase M2 (PKM2), which were significantly associated with HCC. We tested these for risk stratification of HCC in multivariable models adjusted for demographic and clinical variables, as well as batch and site. These markers reflect ongoing biology in the liver. They potentially indicate the presence of HCC early in its evolution and before it is manifest as a detectable lesion, thereby providing a set of markers that may be able to stratify risk for HCC.

## INTRODUCTION

Most HCC is diagnosed late, resulting in poor clinical outcomes [1–4]. Current screening for HCC involves serial ultrasound (US) and alpha-fetoprotein (AFP) that do not have a high sensitivity/specificity, which may be related to the limited non-mechanistic basis of these strategies [5]. Outcomes remain poor using these screening methods [6] and most patients are diagnosed at an advanced stage [1, 3], indicating a failure of early detection. Thus, there is an urgent need for reliable circulating biomarkers that can stratify the risk of developing early HCC. The recently described PLSec [7] and GALAD [8] have not been validated in extensive and well-characterized studies and do not capture ongoing biology in the liver. Collectively, these results support the need for improved screening and surveillance strategies that reflect pathology in the continuum of liver disease from cirrhosis to HCC.

Molecular heterogeneity, multiple etiologies, and divergent pathophysiology associated with HCC have delineated molecular pathways that control the initiation and progression of HCC [9–11]. Etiologies associated with the initiation and progression of HCC include cirrhosis resulting from hepatitis viral infection, exposure to toxins (for example, alcohol or aflatoxin), metabolic syndromes, and mutations in one or more genes (for example, TERT, SMAD4, TP53, and HGF) [12–15]. Additionally, signaling pathways linked to HCC include those involving the mitogen-activated protein kinase (MAPK) cascade, the phosphoinositide 3-kinase (PI3K) cascade, the WNT-β-catenin pathway, the insulin-like growth factor-1 (IGF1) pathway, and the transforming growth factor-β (TGF-β) pathway [16, 17].

Among these pathways, as a regulator of hepatocyte proliferation and tissue regeneration, the TGF-β pathway is an essential link between fibrosis/cirrhosis and the development of HCC. Alterations in the TGF-β pathway members have been observed in nearly 40% of liver and gastrointestinal cancers [18–21]. TGF-β signaling plays a complex context-driven role in liver disease and cancer. It regulates not only hepatocyte proliferation but also fibrosis, functional activity of the stroma including cells of the immune system and stellate cells, genomic stability in the context of genotoxic injury, as well as hepatocarcinogenesis and the tumor microenvironment [12, 22–25]. Furthermore, multiple genetically engineered mouse models with impaired TGF-β signaling are susceptible to HCC [16, 26–28]. Mice heterozygous for *Sptbn1*, encoding the multifunctional Smad3 adaptor protein βII-spectrin (β2SP), alone or when combined with the heterozygosity of *Smad3* (*Sptbn1*^+/−^, *Smad3*^+/−^*Sptbn1*^+/−^) spontaneously develop GI cancers and hepatocellular cancer (HCC) in the C57BL/6J background [13, 21]. Furthermore, haploinsufficiency phenotypes include severe fatty liver disease, fibrosis, inflammation, and early adenomas of the colon, consistent with a premalignant state. The remaining Smad3 or *Smad3* wild-type allele is also lost in the late stages of tumor progression. These data suggest that gene dosage of *Smad3* and *Sptbn1* drives tumorigenesis and that progression involves loss of the wildtype *Smad* allele. Interestingly, βII-spectrin has a liver-specific role in response to the diet-induced fatty liver through interacting with the lipogenic transcription factor SREBP1 [29]. A caspase-mediated cleavage product of βII-spectrin contributes to acetaminophen toxicity in the liver [30], and mice treated with shRNA targeting β2SP show significantly reduced acetaminophen-induced hepatotoxicity [30]. Furthermore, liver-specific knockout of *Sptbn1* (βS-LSKO) or siRNA targeting βII-spectrin reduces diet-induced Metabolic Dysfunction Associated Steatohepatitis (MASH) and protects against HCC [29], suggesting that targeting βII-spectrin in experimental studies might provide new insights into MASH and steatosis-associated HCC. Moreover, these mouse models and mechanistic studies provide an ongoing approach towards identifying biologically important biomarkers that could stratify risk for HCC.

Alterations in the TGF-β signaling pathway could reflect a continuum of fibrosis to cirrhosis to cancer in the liver. Thus, we hypothesize that the TGF-β pathway-enriched biomarkers may serve as biomarkers in the evolution of HCC and stratify patients at risk for HCC. In addition, we hypothesized that the integrated animal model-to-human studies program would yield new TGF-β driven mechanistic biomarkers that could be valuable in yielding additional biomarkers that could stratify the risk of HCC. Here, we identify a 31-biomarker panel, which we refer to as TGF-β based Protein Markers for Early Detection of HCC (TPEARLE), that could potentially stratify cirrhotic patients at high risk for developing HCC. In addition, we found two other biologically relevant markers, Myostatin and Pyruvate Kinase M2 (PKM2), which were significantly associated with HCC.

## RESULTS

### Selection of potential HCC protein markers

Our cohort for the main analysis included 216 patients with cirrhosis, of whom 59 had HCC (18 early stage defined as one tumor <5 cm or </= 3 tumors, each <3 cm, with no extrahepatic spread). Demographics and lab values differed between groups (Table 1). Patients with HCC were older, more often male, more often Black or Asian, more likely to be Child-Pugh class A, and with a cirrhosis etiology more likely to be HCV or HBV. The HCC patients had higher levels of AFP and albumin and lower levels of bilirubin and Alkaline phosphatase (ALP) than those without HCC.

**Table 1:** Patient characteristics in full sample and stratified by presence of HCC

Variable	All patients (*n* = 216)	Cirrhosis without HCC (*n* = 157)	Cirrhosiswith HCC (*n* = 59)	*p*
Age	58 ± 11	57 ± 11	63 ± 7	<0.0001
Sex female	61 (28%)	51 (32%)	10 (17%)	0.02
Race				
Asian	20 (9%)	7 (4%)	13 (22%)	<0.0001
Black	44 (20%)	29 (18%)	15 25%)	
Hispanic	14 (6%)	10 (6%)	4 (7%)	
Pacific Islander	7 (3%)	1 (1%)	6 (10%)	
White	123 (57%)	103 (66%)	20 (34%)	
Other/unknown	8 (4%)	7 (4%)	1 (2%)	
BMI	29 ± 6	29 ± 6	30 ± 6	0.26
ChildPugh Class				
A	89 (41%)	45 (29%)	44 (75%)	<0.0001
B	82 (38%)	70 (45%)	12 (20%)	
C	45 (21%)	42 (27%)	3 (5%)	
Etiology				
HCV	96 (44%)	60 (38%)	36 (61%)	0.003
HBV	10 (5%)	3 (2%)	7 (12%)	0.002
NAFLD	26 (12%)	16 (10%)	10 (17%)	0.17
ALD	82 (38%)	60 (38%)	22 (37%)	0.90
Alpha feto protein (AFP) ng/ml, *median (IQR)*	5.7 (2.7–18.3)	3.9 (2.3–7.8)	15.7 (6.0–108.8)	<0.0001
Bilirubin mg/dL, *median (IQR)*	1.5 (0.8–2.5)	1.8 (1.1–3.6)	0.8 (0.7–1.2)	<0.0001
Serum creatinine mg/dL, *median (IQR)*	0.9 (0.7–1.1)	0.9 (0.7–1.2)	0.9 (0.8–1.1)	0.64
Serum albumin g/dL, *median (IQR)*	3.4 (2.8–4.0)	3.1 (2.7–3.8)	3.7 (3.3–4.2)	<0.0001
ALT U/L, *median (IQR)*	40 (28 – 72)	39 (26–66)	49 (30–94)	0.05
AST U/L, *median (IQR)*	60 (38 – 97)	60 (387–94)	55 (33–118)	0.79
ALP U/L, *median (IQR)*	126 (85 – 173)	136 (95–184)	99 (85 – 144)	0.013

**Figure 1:**
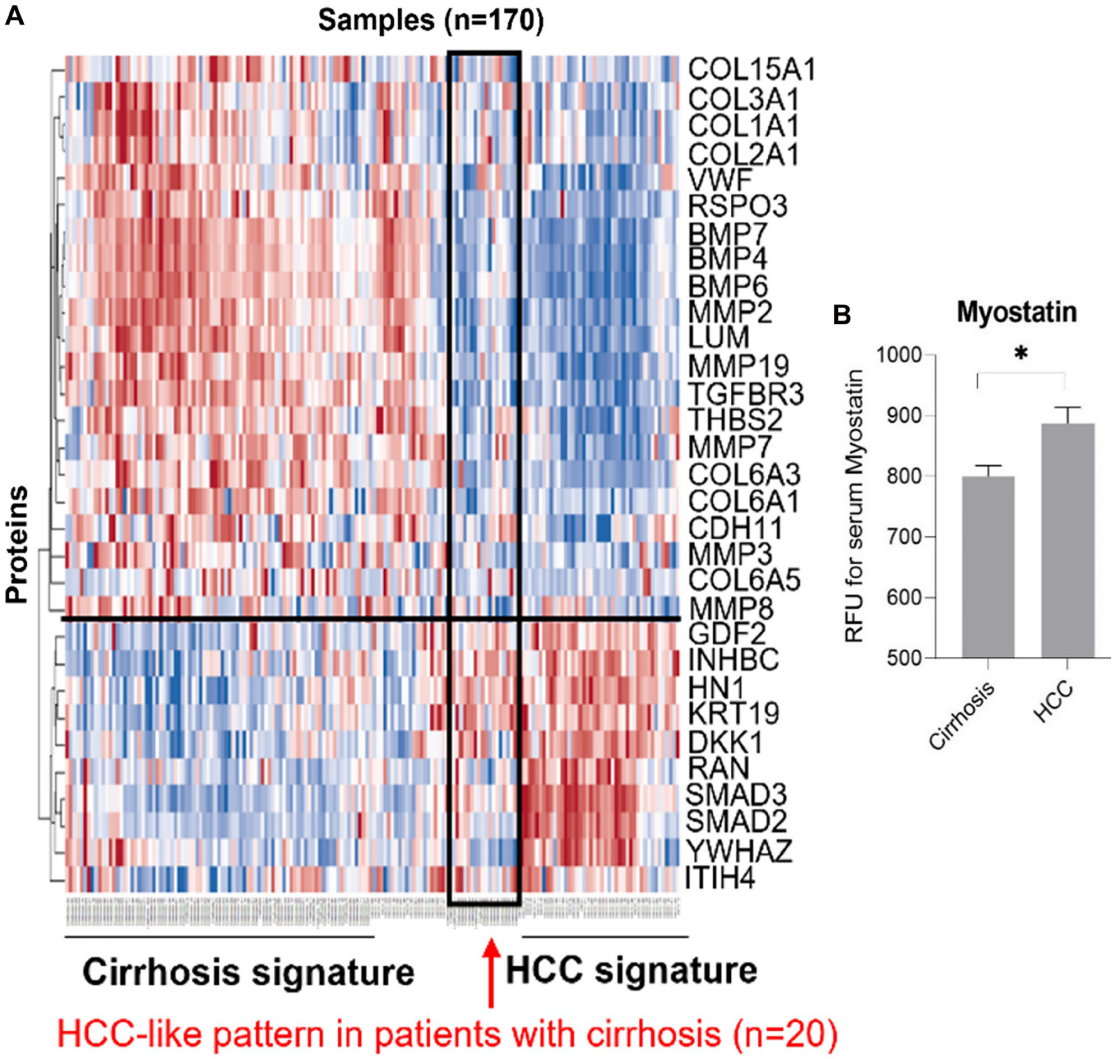
TGF-β pathway-related serum markers stratify HCC risk in cirrhotic patients (TPEARLE). (**A**) Unsupervised clustering analysis of TGF-β pathway-associated proteins in cirrhosis (*n* = 104) and HCC patients (*n* = 66). (**B**) In a larger cohort, serum Myostatin is higher in HCC (*n* = 157), compared to cirrhotic patients (*n* = 380).

**Figure 2:**
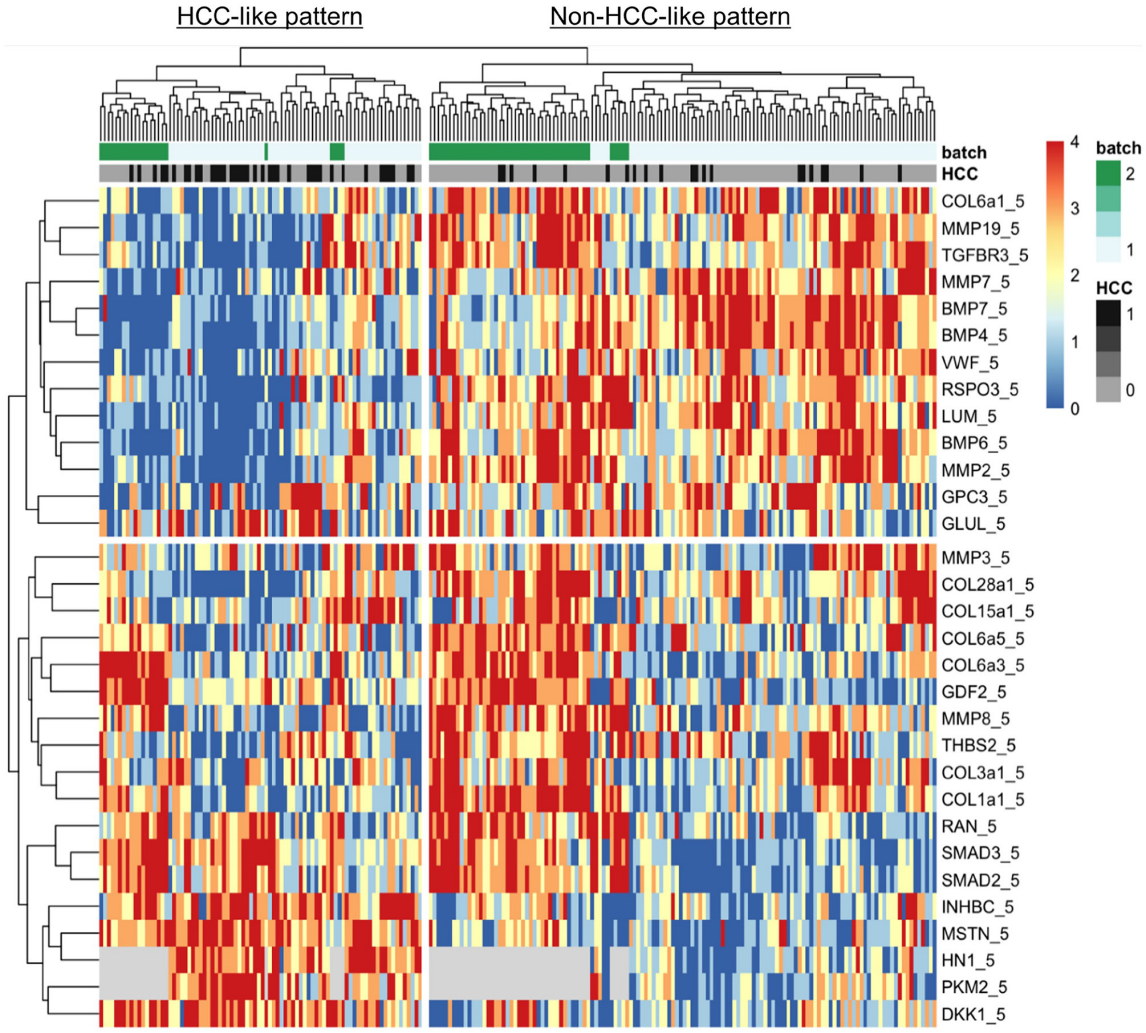
Heatmap showing TGF-β pathway-related serum proteomic markers associated with HCC in cirrhotic patients. Unsupervised k-means clustering of markers and patients *N* = 216 patients, 59 with HCC, using batch 1 and 2 data. Markers are scored as quintiles (0–4). Grey = missing. There are distinct HCC-like and non-HCC-like patterns of marker levels and batch effects.

Our examination of 108 proteins from the TGF-β family members [4, 19] and some known biomarkers for HCC [7, 31–34], after unsupervised clustering of markers and patients using our batch-1 data, revealed a promising proteomic pattern that differentiates cirrhosis from HCC, using an early cohort of *N* = 170 (Figure 1A). Markers positively associated with HCC included INHBC, HN1, DKK1, RAN, SMAD3, SMAD2, and YWHAZ. Markers negatively associated with HCC included COL3a1, BMP7, BMP4, BMP6, LUM, MMP19, TGFBR3, and MMP7. Notably, 20 patients with cirrhosis presented an HCC-like pattern, suggesting that they may be a group at high risk for developing HCC. We refer to the proteomic signature differentiating HCC from non-HCC in patients with cirrhosis as TGF-β based Protein Markers for Early Detection of HCC (TPEARLE). PKM2 and myostatin (Figure 1B), markers known to play a role in HCC, were also significantly associated with HCC in a later cohort of *N* = 467. Including the batch 1 and 2 cohort, the pattern was similar (Figure 2). SMAD2, SMAD3, INHBC, MSTN, HN1, PKM2, and DKK were positive HCC markers (dark red indicating the highest marker quintile, blue indicating the lowest quintile). BMP7, BMP4, COL5a1, MMP19, TGFBR3, MMP7, LUM were negatively associated with HCC.

We and others have focused on refining untargeted circulating proteins unique to HCC as potential biomarkers. In preliminary studies, we identified circulating proteins that are components of the TGF-β signaling pathway in HCC.

### Additional markers PKM2 and myostatin

**Figure 3:**
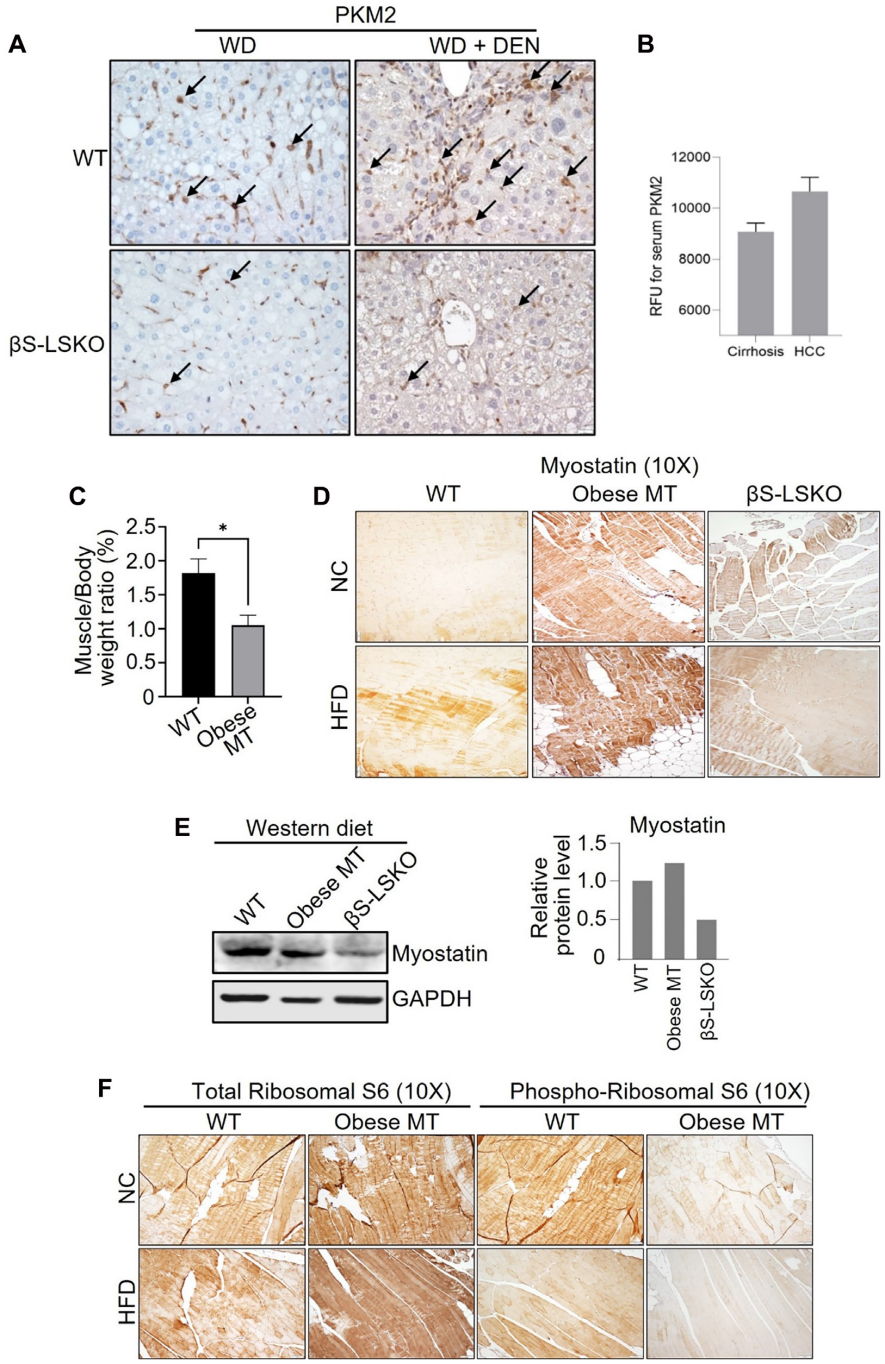
Expression of TGF-β driven mechanistic marker Myostatin and PKM2 in MASH/HCC mouse model. (**A**) Increased PKM2 expression in hepatic Kupffer cells of liver tissue from the MASH/HCC mouse model (Western diet (WD) + Diethylnitrosamine (DEN)), and (**B**) in human HCC. PKM2 expression is significantly reduced in βS-LSKO mice, which block HCC. Arrows indicate positive staining. (**C**–**F**) Muscle loss and increased expression of Myostatin in muscle tissue from obese mouse tissues (Obese MT): (C) muscle/body weight ratio decreased, ^*^p < 0.05; (D, E) Increased Myostatin; (F) decreased p-RPS6/total-RPS6 ratio in muscle tissue from obese mouse.

Lastly, we explored potential new biomarkers to continue examining our mouse models and query our mechanistic studies. We observed that core metabolic pathways, such as glycolysis and gluconeogenesis, were significantly altered, and critical metabolites, such as serine, pyruvate, aconitate, and malate, were substantially increased in obese mouse tissues -which we refer to as Obese MT [29, 35] -that were corrected with blocking SPTBN1 (βS-LSKO) [29]. Transcriptomic analysis shows increased expression of glucose-related metabolic genes involved in the TCA cycle, pyruvate metabolism, pentose phosphate pathway, and serine metabolism in the liver tissues of obese mice, compared to the normal WT group (Supplementary Figure 1). We observed that a key enzyme in glycolysis- PKM2 expression is substantially increased in DEN-induced HCC liver tissues. In contrast, PKM2 expression is significantly reduced in liver tissues of βS-LSKO mice fed on normal chow diet (NC) and Western diet (WD) (Figure 3A). Furthermore, we observed increased PKM2 expression in hepatic Kupffer cells of the MASH-associated HCC mouse model, and the βS-LSKO mice showed a significant reduction in PKM2 expression in these cells (Figure 3A). Indeed, we observed that PKM2 levels are significantly increased in HCC patients compared to cirrhotic individuals (Figure 3B).

Another critical characteristic of cirrhosis and HCC, as well as in our mouse models of HCC, is severe muscle loss [36–38]. We observed that similar to the human fatty liver disease, our obese mice show marked sarcopenia that is corrected in the liver-specific knockout of SPTBN1. Because of its biological functional role as a TGF-β superfamily member and its role in muscle loss, we also examined Myostatin levels in our samples. Compared to the WT mice, the obese mice (obese MT) showed significant muscle loss (Figure 3C) with increased Myostatin expression (Figure 3D, 3E), and decreased levels of p-RPS6/total-RPS6 ratio (indicating mTOR signaling inhibition, Figure 3F) in striated muscle tissue. These changes are associated with the development of sarcopenia during liver cirrhosis and cancer [39, 40]. In contrast, we observed that Myostatin expression is decreased in βS-LSKO tissues (Figure 3D, 3E).

**Figure 4:**
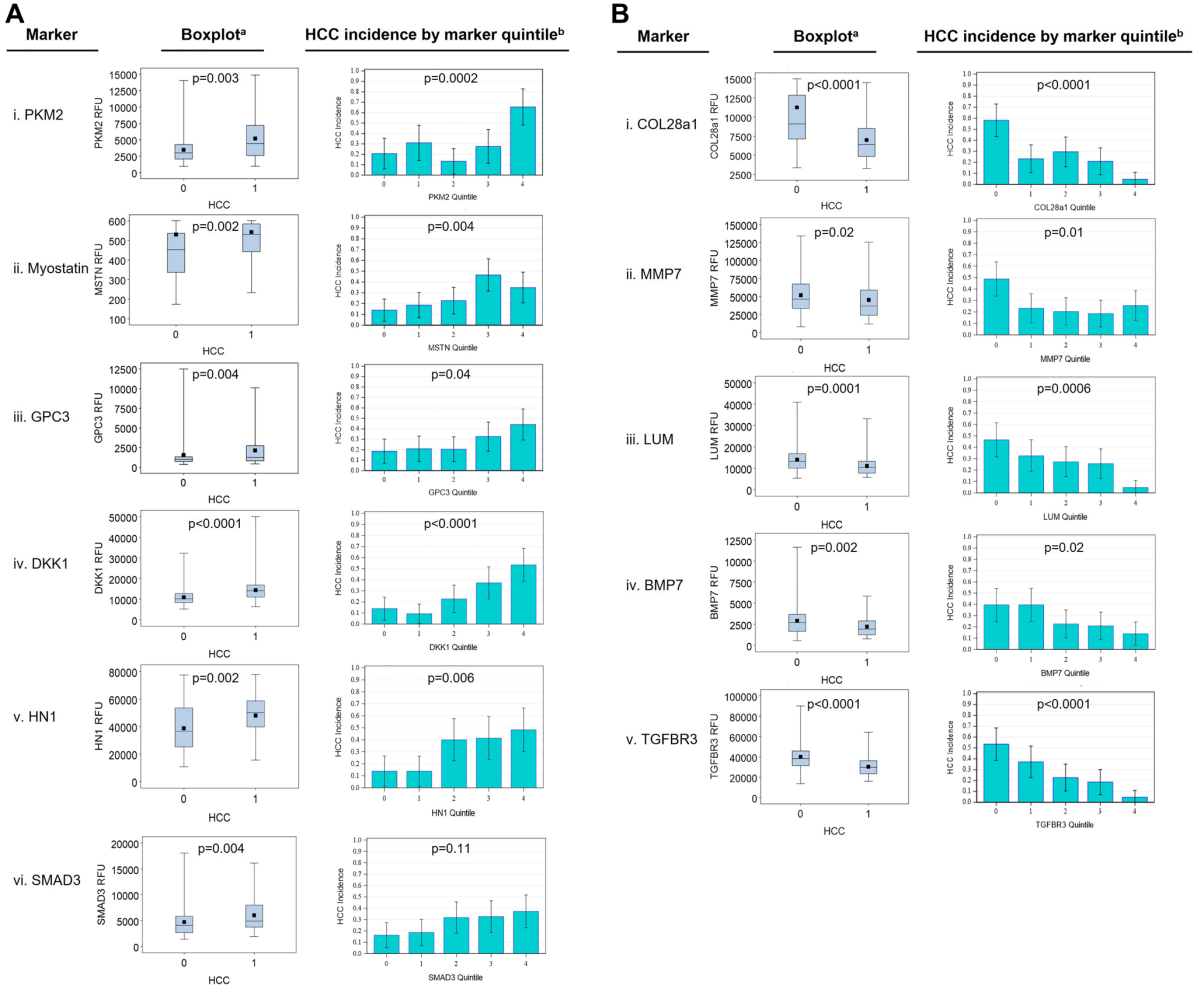
Association of selected marker levels by HCC status. (**A**) Markers positively associated with HCC. (**B**) Markers are negatively associated with HCC. In box plots, the black square is the mean, the horizontal line is the median, the box shows the intra-quartile range, and ‘whiskers’ show the range. In the quintile chart, error bars show the 95% confidence interval. ^a^FDR-adjusted Kruskal-Wallis *p* value, ^b^chi-square *p* value.

Using the 108 markers as well as PKM2 and myostatin (MSTN), in batch 1 and 2 cohorts, we found 31 markers with false discovery rate (FDR) *p* values < 0.10 from univariable Kruskal-Wallis tests for the associations of markers with HCC vs. cirrhosis, which we retained for further analysis (Table 2). Markers positively associated with HCC included PKM2, MSTN, GPC3, INHBC, HN1, DKK1, and SMAD3 (Figure 4A). Markers negatively associated with HCC included COL28A1, TGFBR3, BMP7, LUM, and MMP7 (Figure 4B).

**Table 2:** Univariable and multivariable *p* values for 31 proteomic markers

Marker	Univariable *p* values			Multivariable model	
	Raw	Bonferroni	False discovery rate	Adjusted OR (95% CI)	*p*
MMP19	<.0001	<.0001	<.0001	0.62 (0.42–0.92)	0.02
TGFBR3	<.0001	<.0001	<.0001	0.53 (0.35–0.80)	0.002
DKK1	<.0001	<.0001	<.0001	1.73 (1.20–2.49)	0.0035
MMP2	<.0001	<.0001	<.0001	0.62 (0.43–0.90)	0.01
COL28A1	<.0001	<.0001	<.0001	0.48 (0.30–0.76)	0.002
BMP6	<.0001	<.0001	<.0001	0.60 (0.40–0.91)	0.015
INHBC	<.0001	0.0001	<.0001	1.41 (1.00–2.00)	0.051
BMP4	<.0001	0.0010	0.0001	0.58 (0.39–0.86)	0.006
RSPO3	<.0001	0.0011	0.0001	0.61 (0.42–0.89)	0.009
LUM	<.0001	0.0014	0.0001	0.53 (0.35–0.81)	0.003
COL1A1	0.0003	0.0080	0.0007	0.87 (0.58–1.30)	0.49
COL6A1	0.0003	0.0104	0.0009	0.75 (0.54–1.04)	0.09
BMP7	0.0009	0.0273	0.0020	0.52 (0.34–0.79)	0.002
MSTN	0.0009	0.0286	0.0020	1.64 (1.14–2.35)	0.007
HN1	0.0010	0.0301	0.0020	1.68 (1.11–2.54)	0.014
MMP8	0.0012	0.0369	0.0023	0.71 (0.48–1.05)	0.09
PKM2	0.0014	0.0443	0.0026	1.58 (1.02–2.44)	0.04
SMAD3	0.0021	0.0661	0.0037	1.60 (1.02–2.49)	0.04
GPC3	0.0026	0.0800	0.0042	0.89 (0.59–1.33)	0.56
COL6A5	0.0035	0.1082	0.0054	0.68 (0.46–1.00)	0.053
VWF	0.0083	0.2588	0.0119	0.87 (0.61–1.24)	0.43
COL15A1	0.0084	0.2607	0.0119	0.62 (0.42–0.91)	0.015
COL3A1	0.0132	0.4087	0.0178	0.99 (0.68–1.43)	0.94
COL6A3	0.0160	0.4957	0.0207	0.84 (0.52–1.34)	0.45
MMP7	0.0198	0.6144	0.0246	0.52 (0.35–0.78)	0.0015
MMP3	0.0340	1.0000	0.0406	0.89 (0.64–1.24)	0.49
THBS2	0.0391	1.0000	0.0449	0.77 (0.54–1.09)	0.14
SMAD2	0.0631	1.0000	0.0681	1.33 (0.88–2.03)	0.18
GDF2	0.0637	1.0000	0.0681	1.49 (0.94–2.37)	0.09
GLUL	0.0678	1.0000	0.0700	1.17 (0.80–1.71)	0.42
RAN	0.0727	1.0000	0.0727	1.26 (0.85–1.86)	0.25

We used univariable Kruskal-Wallis test with Bonferroni and False Discovery Rate adjustment for multiple testing. The multivariable model adjusted for demographic variables, lab test values, batch, and site, with markers coded as quintiles. The markers with significant multivariable results are the most promising and should go on to further testing.

Potential confounding variables that were significantly associated with HCC in the clinical multivariable model included age (*p* = .002), HCV (*p* = .008), HBV (*p* = .0005), log10AFP (*p* < .0001), log10ALT (*p* = .04), site (*p* < .0001), and batch (*p* = .003). After adjusting for these, we found 16 markers with significant independent associations with HCC (Table 2). Based on adjusted OR (aOR), the strongest positive HCC markers adjusted for confounding factors were: DKK1 (aOR 1.73), HN1 (aOR 1.68), MSTN (aOR 1.64), SMAD3 (aOR 1.60), and PKM2 (aOR 1.58). The strongest negative markers for HCC were COL28a1 (aOR 0.48), BMP7 (aOR 0.52), MMP7 (aOR 0.52), TGFBR3 (aOR 0.53), and LUM (aOR 0.53) (Table 2). Other significant markers included MMP19, MMP2, BMP6, BMP4, RSPO3, and COL15a1.

## DISCUSSION

A fundamental hypothesis we sought to test was whether biomarkers from the TGF-β signaling pathway might be of novel value in risk stratification of HCC in the clinical cirrhotic setting. We observe that in this cohort of patients with cirrhosis, alterations in the serum levels of members of TGF-β protein family may be promising markers for early detection of HCC [41]. We found 16 such markers significantly associated with HCC after adjusting for potentially confounding variables. These proteins are promising as potential additional markers that could be used in the surveillance of patients at risk for HCC along with AFP and ultrasound. These markers can be compiled with other published markers to develop a panel of markers predictive of HCC using small volumes of serum. TGF-β signaling initiates context-dependent responses and is critical for protein homeostasis (proteostasis) [22, 23].

Metabolic alterations are a major contributing factor in the progression of diseases in fatty liver disease to cancer. Earlier, we had observed a liver-specific role of the Smad3 adaptor βII-spectrin in response to the diet-induced fatty liver through its interactions with the lipogenic transcription factor SREBP1 [29]. Moreover, liver-specific knockout of *Sptbn1* (βS-LSKO) or siRNA targeting βII-spectrin reduces diet-induced MASH and protects against HCC [29], suggesting that targeting βII-spectrin may be a viable strategy to better understand mechanisms, as well as identify new biomarkers of MASH and steatosis-associated HCC and effects on restoration of metabolic function [29]. Therefore, we examined multiple enzymes involved in glycolysis. Interestingly, we observed that PKM2 expression is substantially increased in DEN-induced HCC liver tissues. In contrast, PKM2 expression is significantly reduced in liver tissues of βS-LSKO mice. Enzymes such as PKM2 that catalyze the last step of glycolysis to generate pyruvate and ATP serve as “gatekeepers” of metabolic flux from glycolysis to the tricarboxylic acid cycle (TCA cycle). Importantly, mutually exclusive alternative splicing of exons 9 and 10 in the PKM gene leads to the expression of either the PKM1 or PKM2 isoform. PKM2 is overexpressed in human HCC and also in chemically induced mouse HCC [42, 43]. These studies suggest that PKM2 could represent an additional TGF-β driven mechanistic marker for risk stratification of HCC.

Cirrhosis and HCC are characterized by severe muscle loss- sarcopenia [36–38]. Myostatin, a TGF-β superfamily member expressed primarily in skeletal muscle, inhibits muscle growth and causes sarcopenia in cirrhosis [39, 44–49]. Muscle-specific deletion of the *myostatin* (myostatin^MSD^) gene prevents hepatic steatosis with high-fat diet (HFD) in mice [50]. Because of its biological functional role as a TGF-β superfamily member, we also examined Myostatin levels in our samples. We also observed that, similar to human fatty liver disease, obese mice show marked sarcopenia with increased Myostatin levels that are corrected in the liver-specific knockout of SPTBN1. These studies reflect that these are biologically functional circuits that could provide new serum markers for risk stratification of HCC.

A limitation of this study is the size of the patient cohort and the number of cases with HCC. The major strengths of this study, however, include the use of a set of markers with known mechanistic roles in HCC tumor formation and an approach that examines their association with HCC by first using unsupervised machine learning methods, and then by use of using methods that account for skewed marker distributions, multiple testing, and the potential for confounding by other variables that are associated with HCC. These results reflect the discovery of new and potentially important blood-based markers that show promise in their ability to provide early detection and risk stratification of HCC.

## MATERIALS AND METHODS

### Cohort analysis

The cohort of cirrhosis patients (216 patients) was recruited from five medical centers in the United States (George Washington University-GW, University of Maryland -UM, University of Hawaii -UH, Northwell Health -NH and University of California at Los Angeles -UCLA), using two batches of samples, designated batch 1 and 2. Cirrhosis was diagnosed using either FibroSure/FibroTest >0.74, AST to Platelet Ratio Index (APRI) >2 [51], or Fibrosis-4 (FIB-4) >3.25 [52], or with histological/ imaging evidence of cirrhosis, or vibration controlled transient elastography (VCTE) >12.5 kPA or clinical evidence of portal hypertension. HCC diagnosis was confirmed by histology or using contrast-enhanced imaging (CT or MRI) showing Liver Reporting and Data System (LI-RADS^®^) 5/Organ Procurement Transplant Network 5 lesions (https://www.acr.org/Clinical-Resources/Reporting-and-Data-Systems/LI-RADS). All HCC patients were treatment naïve. Controls were designated as those with cirrhosis, without HCC at enrollment, and on an imaging study 6 months after enrollment. Because we focused on detecting models that might be clinically useful in a patient population with cirrhosis under surveillance for HCC, we chose not to include a normal control group. In support of this approach, several studies have shown that models using normal controls are over-optimistic, can be misleading, and require different controls to define a more realistic clinical setting [53–55].

We compared patients with vs. without HCC demographic and clinical variables using chi-square for categorical variables and either a *t*-test or Kruskal-Wallis test for continuous variables, depending on whether they were normally distributed or skewed.

### SomaScan analysis

We collaborated with SomaLogic, a Biotech company focused on precision health, for serum proteome analyses [56–59]. SomaScan assay utilizes a small volume of 130 μl serum or plasma with a readout of ~5000 proteins, potentially including multiple liver cancer biomarkers. Samples were analyzed in two batches. In this study, we focused on 108 markers that included members of the TGF-β pathway. Raw proteomic data were used in units of relative fluorescence units. Only one aptamer per protein was used for proteins with multiple aptamers, selecting the one with the strongest association with HCC.

We used unsupervised k-means clustering of subjects and proteins to produce a heat map showing clusters of patients with similar patterns of protein levels, using the R pheatmap and ComplexHeatmap packages. When the patient clustering corresponded to those patients with vs. without HCC, it suggested that the protein pattern differentiating this patient group was predictive of HCC. This group of proteins was then explored further, as described below.

To examine the distribution of marker levels in patients with vs. without HCC, we examined boxplots. Since most markers had skewed distributions, we used the non-parametric Kruskal-Wallis test to associate marker level with HCC status. Then we used false-discovery-rate (FDR) adjusted *p* values to account for multiple testing. Markers with FDR *p* < 0.10 were considered for further study. We coded these markers into quintiles to eliminate skewness and to examine the functional relationship of marker level with the incidence of HCC using chi-square test.

We next examined each marker’s association with HCC after adjusting for the demographic and clinical variables that differed between HCC and non-HCC patients. These baseline variables could have acted as confounding variables, leading to differences in marker levels due to the baseline variables, rather than HCC. Coding the proteomic markers as quintiles also had the effect of equating the variances of the markers so that their effect sizes (i.e., their adjusted odds ratio with HCC after adjusting for covariates in the multivariate logistic regression model) could be compared with each other to determine which had the strongest effect. To examine the independent association of each marker with HCC after adjusting for potential confounding variables, we used one multiple logistic regression model per marker with demographic and clinical variables that had univariable *p* < 0.10 used as covariates. The markers that had significant independent effects in this model were considered to be potentially useful markers that deserved further study. We used SAS (version 9.4, Cary, NC) and R (ComplexHeatmap and pheatmap packages) for data analysis, with *p* < .05 considered significant, unless otherwise specified.

### Animal models

All mice utilized in the study originated from a C57BL/6 background and are considered suitable for studies in obesity and cancer [29, 60–62]. To induce liver steatosis through a high-fat diet (HFD), male and female mice aged 10 to 12 weeks were subjected to either the normal chow diet (NC) or HFD (ENVIGO, catalog no. TD.06414) for durations spanning 12 to 28 weeks. In the case of MASH-associated HCC induced by a combination of Diethylnitrosamine (DEN) and a Western diet (WD), mice were administered weekly injections of DEN (50 mg/kg) for two consecutive weeks, followed by a one-week pause, amounting to 6 cycles over an 18-week period. The WD diet was introduced one week after the initial DEN injection, continuing for 21 to 22 weeks in total.

### Immunoblotting analysis

For Western blot analysis of Myostatin levels in muscle, skeletal muscle from WT, obese mouse (Obese MT), and βS-LSKO mouse tissues were homogenized with 250 μL of Tissue cell lysis buffer (GOLDBIO, Cat. GB-181) containing protease inhibitor and phosphatase inhibitor cocktails (Millipore-Sigma) on ice using a pellet pestle motor homogenizer. After homogenization, samples were gently rotated on a rotator at 4°C for 40 min. Samples were vortexed and then centrifuged at 16000 g in a microcentrifuge at 4°C for 20 min to pellet insoluble material. Protein concentration was determined using the Bio-Rad protein assay dye reagent, following the manufacturer’s instructions (DC protein assay reagent A and B; #5000113, #5000114). Fifty micrograms of protein were then separated by SDS-PAGE and transferred to nitrocellulose Membrane (Bio-Rad, #1620112). Immunoblotting was performed using the following antibodies: Myostatin (Proteintech, Cat. 19142-1-AP) and GAPDH (Santa Cruz, sc-32233). ImageJ software (version 1.53t, National Institutes of Health) evaluated and quantified the protein bands.

### Immunohistochemical analysis

For immunohistochemical analyses, mouse tissues were fixed in 10% formalin and embedded in paraffin (FFPE) following standard procedures. FFPE sections were deparaffinized, hydrated, and pretreated for antigen retrieval in 10 mM citrate buffer, pH 6.0 (Vector Laboratories, Cat. H-330). Endogenous peroxidase activity was blocked with 3% H_2_O_2_ solution for 30 min. Nonspecific binding was blocked with 10% normal goat serum (Vector Laboratories) in 0.1% Triton X-100 in a 1× PBS solution (PBST) for 1 hr at room temperature in a humidifying chamber. Sections were labeled with antibodies specific to PKM2 (Cell signaling, #4053), Myostatin (Proteintech, Cat. 19142-1-AP), phospho-S6 Ribosomal (Cell signaling, #2215S), and S6 Ribosomal Protein (Cell signaling, #2217T) overnight at 4°C in the humidifying chamber. After being washed with 1× PBST three times for 3 min, sections were incubated with biotinylated secondary antibodies (Vectastain Elite ABC kit, PK6106) for 30 min at room temperature in the humidifying chamber. Finally, the sections were stained using DAB kit (Dako EnVision Dual link System HRT, K4065) for signal amplification and detection.

## SUPPLEMENTARY MATERIALS



## Abbreviations

HCC: hepatocellular carcinoma
TGF-β: transforming growth factor β
PKM2: Pyruvate Kinase M2
βS-LSKO: liver-specific knockout of *Sptbn1*
TPEARLE: TGF-β based Protein Markers for Early Detection of HCC
NC: normal chow diet
HFD: high-fat diet
WD: Western diet
DEN: Diethylnitrosamine
