# Supplementary material for: Mechanistically based blood proteomic markers in the TGF-β pathway stratify risk of hepatocellular cancer in patients with cirrhosis
Source: Genes Cancer. 2024 Feb 1;15:1–14. doi: 10.18632/genesandcancer.234 (PMC10843195; doi:10.18632/genesandcancer.234)
Supplement: Supplementary file 1 [file ganc-15-234-s001.pdf]

Mechanistically based blood proteomic markers in the TGF-β pathway stratify risk of hepatocellular cancer in patients with cirrhosis

SUPPLEMENTARY MATERIALS

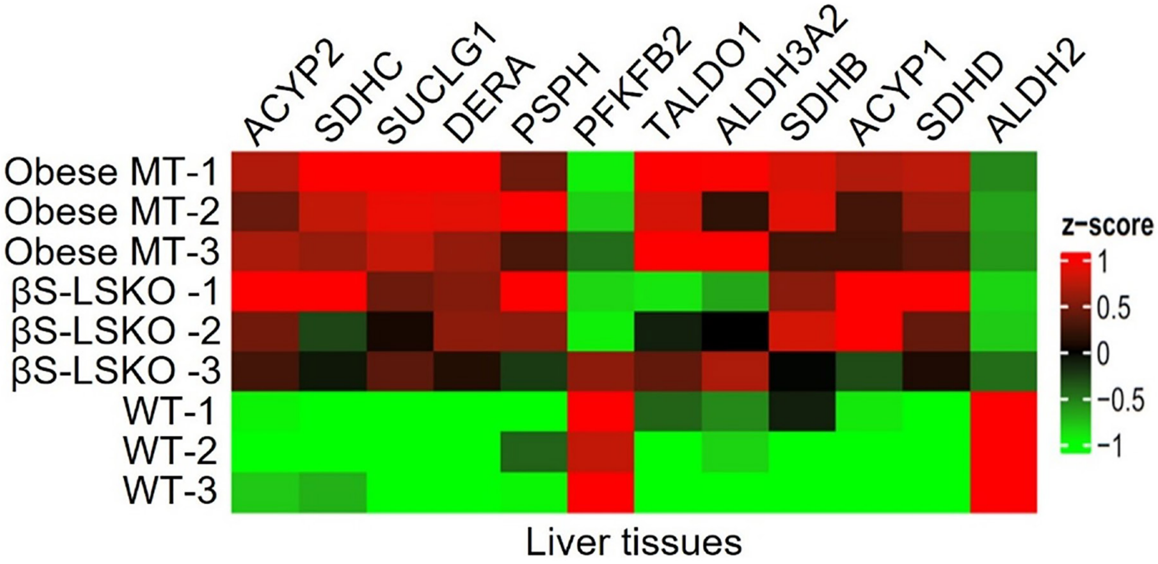

Supplementary Figure 1: Altered glucose metabolic processes in the liver tissues from obese mice. Heatmap indicate relative expression level of glucose metabolic genes in liver tissue from mouse via RNA-seq analysis in the mentioned groups. Abbreviations: WT: Wild type; Obese MT: Obese mouse tissues; βS-LSKO: liver-specific knockout of *Sptbn1*. ($p < 0.05$).
